# Study of Styrene Butadiene Rubber Reinforced by Polybutadiene Liquid Rubber-Modified Silica

**DOI:** 10.3390/polym16202866

**Published:** 2024-10-10

**Authors:** Qing Liao, Xiao Tang, Jiao Tang, Jiaxiang Tang, Housheng Xia, Zhongyi Sheng, Jianping Zhou, Junfeng Niu

**Affiliations:** 1 School of Biological and Chemical Engineering, Zhejiang University of Science and Technology, Hangzhou 310023, China; 2 Zhejiang Xianghe Railway Fastener Research Institute, Zhejiang Tiantai Xianghe Industrial Co., Ltd., Taizhou 317200, China

**Keywords:** reinforcement, styrene butadiene rubber, silica, liquid rubber, mechanical property, composite materials

## Abstract

The dispersion of silica in rubber systems and its interaction with rubber are two key factors in the preparation of rubber composites with excellent properties. In view of this, silica modified with terminal isocyanate-based polybutadiene liquid rubber (ITPB) is used to improve the dispersion effect of silica in rubber and enhance its interaction with the rubber matrix to improve the rubber’s performance. The impact of different modification conditions on the dispersion of silica and the properties of modified silica-filled rubber composites were studied by changing the amount of ITPB and the modification method of silica, including blending and chemical grafting. The experimental results show that ITPB is successfully grafted onto silica, and the use of modified silica improves the cross-linking density of rubber, promotes the rate of rubber vulcanization, and overcomes the shortcomings of the delayed vulcanization of silica itself. When the ratio of ITPB liquid rubber to silica equals 1:20, the comprehensive performance of rubber is the best, the ITPB-modified silica has a better dispersion effect in rubber, and the rolling resistance is slightly improved, with tensile strength reaching 12.6 MPa. The material demonstrates excellent overall performance and holds promise for applications in the rail, automotive, and electrical fields.

## 1. Introduction

Rubber is extensively utilized across various domains, including automotives, construction, medical devices, and everyday consumer products, owing to its remarkable elasticity, wear resistance, and anti-aging properties [1,2,3,4]. It is employed in the manufacture of tires, seals, shock absorbers, conveyor belts, and various medical and industrial products. To enhance the practical value of rubber, reinforcing agents are often incorporated to meet performance specifications. Among these, precipitated silica plays a pivotal role in reinforcing rubber [5,6,7]. Silica enhances the wear resistance, mechanical strength, and heat aging resistance of rubber [8,9]. Compared to the widely used carbon black, silica can mitigate adverse environmental impacts [10,11]. For rubber composites, the dispersion of fillers and their interaction with the rubber matrix are critical determinants of both dynamic and static mechanical properties [12,13,14,15]. The surface of silica, bearing hydroxyl groups, exhibits a degree of polarity, which adversely affects its compatibility with non-polar rubbers such as styrene–butadiene rubber (SBR) [16,17,18]. The hydrogen bonding between hydroxyl groups leads to stronger interactions among silica particles than between the silica and the rubber matrix, leading to suboptimal dispersion within the rubber [19,20,21]. To enhance silica’s compatibility and dispersion within rubber, the polymeric surface modification of silica is employed. Grafting polymer chains onto the silica surface increases its steric hindrance and organic affinity. The substantial steric hindrance effectively suppresses silica agglomeration, while the enhanced organic affinity improves its dispersion within the polymer matrix [22,23]. Key factors influencing silica dispersion include the length of the grafting modifier chains, the density of polymer grafting, and the compatibility with the matrix. The chemical bonding between grafted polymer chains, such as liquid rubber, and the rubber matrix significantly enhance mechanical performance.

Liquid rubber is an excellent modifier for silica, and many studies have reported on different types of liquid rubber used for modifying rubber fillers [24,25,26,27]. The grafting of liquid rubber molecules, which are similar to the rubber matrix in terms of chemistry, onto silica endows the filler with a strong affinity to the matrix and can also participate in the rubber vulcanization process. Functionalized liquid rubber-modified silica show superior dispersion performance in a rubber matrix compared with silane coupling agents [28]. Among the factors affecting silica dispersion, polymer graft density is the most critical, while the grafting method and the reactivity of the surface hydroxyl or other substituents determine the graft density of the liquid rubber. Chang et al. [29] successfully grafted functionalized liquid isoprene rubber (FLIR) onto silica via blending to improve the interaction between silica and natural rubber and enhance silica dispersion. Although this method is convenient, it is challenging to control the reaction conditions for liquid rubber, which affect the modification efficacy. M. R. Pourhossaini et al. [22] attached hydroxyl-terminated polybutadiene (HTPB) onto the surface of silica nanoparticles using toluene diisocyanate (TDI), aiming to reduce filler–filler interactions and improve silica dispersion in rubber. Despite TDI’s high reactivity, the steric effects of TDI can reduce the activity of the other isocyanate group after reacting with the hydroxyl group, thereby decreasing the grafting efficiency. Sun et al. [30] improved silica dispersion by introducing amino functional groups at the middle and end of the SSBR chains. They compared the reinforcing effects of amino groups located at different positions on the rubber. The results indicated that the incorporation of amino groups onto the SSBR chain indeed improved the dispersion of silica and concurrently enhanced the mechanical properties of the rubber. However, in contrast to modifying silica, the synthesis of SSBR and the grafting of amino functional groups involve a more complex process, incur higher costs, and present challenges for large-scale production. Additionally, epoxy-terminated or hydroxyl-terminated liquid polybutadienes have also been employed for silica surface modification [31,32,33,34]. This study utilizes liquid polybutadiene rubber (ITPB) with small molecules containing highly reactive isocyanate functional groups as the primary material. The superior reactivity of ITPB compared to other isocyanate-functionalized compounds facilitates its grafting onto liquid rubber. Additionally, the molecular characteristics of polybutadiene confer excellent processing capabilities and compatibility with other rubber types. Notably, we observed a scarcity of research in this area. In our experiments, toluene was employed as a solvent to graft ITPB onto the surface of silica particles. The ITPB interacts with hydroxyl groups on the silica surface, effectively reducing its hydrophilicity and preventing particle aggregation. Simultaneously, the ITPB grafted on the silica surface participates in the vulcanization of the rubber matrix through its double bonds, thereby enhancing the dispersion of silica within the SBR matrix and improving the interaction between silica and the matrix, ultimately achieving reinforcement.

## 2. Materials and Methods

### 2.1. Materials

Styrene–butadiene rubber (SBR), precipitated silica, and isocyanate-terminated polybutadiene (ITPB) with a molecular weight of 3000 were purchased from Shenzhen Hongyuan Chemical New Materials Technology Co., Ltd. (Shenzhen, China) Toluene. All materials were of analytical grade, along with other components, such as 1,2-dihydro-2,2,4-trimethylquinoline (RD), zinc oxide (ZnO), and sulfur (S), which were obtained as industrial-grade products from commercial suppliers.

### 2.2. Preparation of ITPB-Modified Silica

Prior to the reaction, the silica must be activated by drying at 120 °C for 2 h. A precise quantity of precipitated silica is placed into a 250 mL three-neck round-bottom flask, followed by the addition of 50 mL of toluene. The mixture is magnetically stirred for 30 min and then subjected to ultrasonic dispersion for 15 min to ensure uniform distribution. Separately, in a clean beaker, 30 mL of toluene is combined with the specified amount of isocyanate-terminated polybutadiene (ITPB), and the solution is stirred until the ITPB is fully dissolved. Nitrogen gas is then introduced into one neck of the 250 mL three-neck flask. The toluene dispersion of silica and the toluene solution of ITPB are added to the flask in accordance with the weight ratios specified in Table 1. The mixture is stirred and maintained at 80 °C for 2 h to facilitate the reaction. Subsequently, the product is centrifuged, and the supernatant is discarded. The residue is washed three times with toluene, with centrifugation and supernatant removal following each wash. The centrifuged product is then dried and subjected to Soxhlet extraction with toluene for 24 h. Finally, the product is vacuum-dried at 80 °C for 6 h, yielding three samples of ITPB-modified silica, designated as SiO_2_-P2, SiO_2_-P5, and SiO_2_-P10, based on the proportion of ITPB added.

### 2.3. Preparation of Silica/SBR Composites

Styrene–butadiene rubber (SBR) with the model number of 1205 was processed on a two-roll mill until a uniform sheet formed. The preprepared ITPB-modified silica was then incorporated into the rubber matrix and mixed for 5 min. Subsequently, zinc oxide, stearic acid, accelerators, carbon black 774, sulfur, and a super accelerator were added sequentially. The mixture was thoroughly blended to achieve uniformity and then the mill opening was set at 0.5 mm and the rubber was passed through the roll several times. It was essential that the whole plastication and mixing process was consistent across all samples. After mixing, the rubber sheet produced with the desired thickness was allowed to stand for 24 h. The curing characteristics of the compounded rubber were assessed using a rheometer to determine the optimal cure time, t90. The vulcanized rubber compound was molded on a flat vulcanizing machine at 160 °C and 15 MPa for t90 + 3 min. The samples were named according to the formulations detailed in Table 2.

### 2.4. FTIR Spectroscopy Analysis

To characterize the molecular composition of silica particles modified with different proportions of liquid rubber, this study employed a Vertex 70 Fourier Transform Infrared (FTIR) Spectrometer from Tianjin Jinbeier Company (Tianjin, China). During the testing process, samples were prepared using the KBr pellet method. Each spectrum was scanned 64 times at a high resolution of 4 cm^−1^ to ensure the accuracy and reliability of the data.

### 2.5. Thermogravimetric Analysis (TGA)

Thermogravimetric Analysis (TGA) provides data on the thermal weight loss of samples, thereby their thermal properties can be elucidated and their structural composition can be inferred. This study utilized the STA449F3 Thermogravimetric Analyzer (TGA) from Netzsch, Selb, Germany, to conduct TGA testing on both unmodified and modified silica particles. All tests were performed under nitrogen (N_2_) atmosphere, with temperatures ramping from room temperature to 800 °C at a heating rate of 15 K/min.

### 2.6. BET Surface Area Analysis

There are numerous pores on the surface of silica particles and surface modification can change their specific surface area. Thus, the changes in specific surface area after modification, measured using the BELSORP-mini II Surface Area Analyzer (Micromeritics, Norcross, GA, USA), can be used to infer the grafting density of liquid rubber on the silica surface. Prior to testing, the silica were degassed at 90 °C and the analysis was conducted under a nitrogen atmosphere.

### 2.7. Scanning Electron Microscopy (SEM)

We utilized the SU1510 Scanning Electron Microscope (SEM) produced by Hitachi (Tokyo, Japan) to observe the microstructure of both unmodified silica particles and silica particles modified with varying proportions of liquid rubber. During sample preparation, the specimens were uniformly coated with a conductive adhesive and subjected to a gold sputtering treatment to enhance surface conductivity and improve imaging quality.

### 2.8. Analysis of Rubber Curing Characteristics

After standing for 24 h, the sample was weighed out to 6 to 10 g for the analysis of curing characteristics. The upper and lower molds of the apparatus (TM2101-T7) were preheated to 160 °C. Upon completion of the preheating process, the sample was placed into a non-rotating vulcanizer to measure parameters such as MH, ML, and t90.

### 2.9. Crosslinking Density

The vulcanized rubber samples were cut into 1 × 1 cm squares with a thickness of 2 mm. These samples were immersed in 100 mL of xylene solvent for swelling. The initial weight was recorded as m0. Every 12 h, the rubber samples were removed, the xylene was quickly wiped from the surface with a paper, and the weight at that moment was recorded. When the difference in weight between successive measurements was less than 0.01 g, the sample was considered to have reached swelling equilibrium, and the weight at this point was recorded as m1. Subsequently, the samples were dried in a vacuum oven and the dry weight was recorded as m2. The crosslink density was calculated using the Flory–Rehner equation.

### 2.10. Mechanical Properties

The compounded rubber was placed into a heat press molding machine and cured at 160 °C. Rubber specimens were obtained after pressing for a duration of t90 + 3 min. The samples were cut into dumbbell-shaped strips with a gauge length of 25 mm, a width of 6 mm, and a thickness of 2 mm. A computer-controlled Universal Testing Machine was used to perform tensile testing at a rate of 500 mm/min. The tensile strength, breaking force, tensile modulus, and elongation at break of the rubber strips were measured and recorded.

### 2.11. Dynamic Mechanical Analysis (DMA)

Dynamic Mechanical Analysis (DMA) was utilized to assess the relationship between the mechanical properties of viscoelastic materials and time, temperature, or frequency. In this study, the dynamic thermomechanical properties of the rubber strips were measured using a DMA (Q800, TA Instruments, New Castle, DE, USA). The testing was conducted over a temperature range of −60 °C to 60 °C, with a heating rate of 3 °C/min and a tensile strain of 0.1%. The frequency of the test was set at 10 Hz.

## 3. Results

### 3.1. Characterization of ITPB-Modified Silica

#### 3.1.1. FTIR Analysis

**Figure 1:**
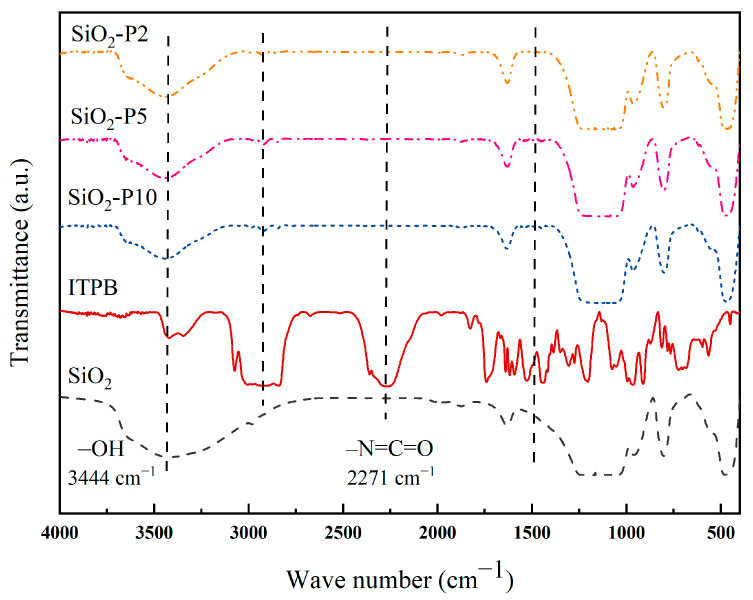
FTIR spectra of unmodified and modified silica.

Infrared spectroscopy was employed to analyze silica, isocyanate-terminated polybutadiene (ITPB), and self-prepared ITPB-modified silica particles. The FTIR spectra revealed variations in the intensity of surface hydroxyl groups and the presence of methylene groups linked with the grafted liquid rubber, both before and after the modification of the silica. The absorption peaks observed in the infrared spectra are depicted in Figure 1.

In the FTIR spectrum of unmodified silica, the pronounced and broad absorption band at 1106 cm^−1^ was attributed to the asymmetric stretching vibration of Si-O-Si bonds, while the broad peak at 3444 cm^−1^ corresponded to the asymmetric stretching vibration of structural water–OH groups. This spectrum is consistent with the standard silica profile. In the FTIR spectrum of isocyanate-terminated polybutadiene (ITPB), the absorption peak near 2926 cm^−1^ was associated with the methylene groups in the liquid rubber, and the prominent peak at 2271 cm^−1^ was attributed to the stretching vibration of the -NCO groups. For the ITPB-modified silica particles, the absorption peak at around 3444 cm^−1^ was significantly diminished, indicating a reaction between the hydroxyl groups on the silica and the isocyanate groups on ITPB. Furthermore, the absence of an absorption peak near 2270 cm^−1^ suggests that the NCO groups of ITPB reacted completely with the silica surface, with any unreacted liquid rubber being removed during extraction. The persistence of a characteristic methylene absorption peak near 2926 cm^−1^ confirms the successful grafting of ITPB onto the silica surface.

**Figure 2:**
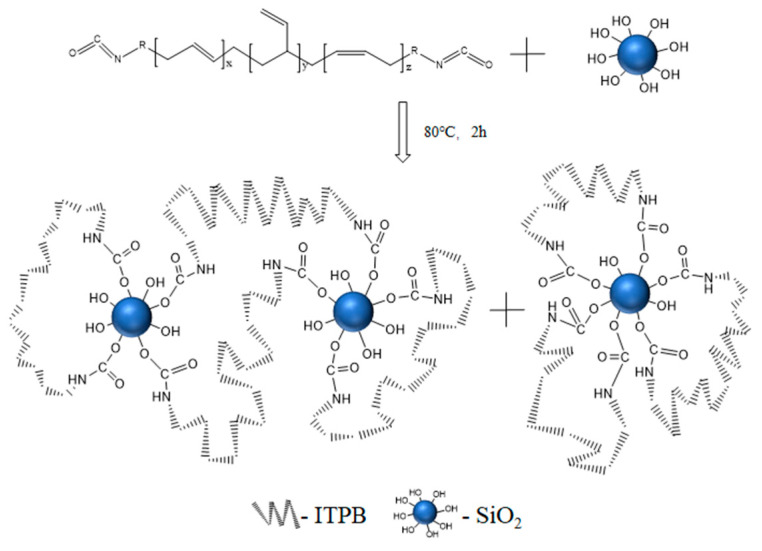
Reaction mechanism of ITPB-modified silica.

The reaction between the hydroxyl groups on the silica surface and the isocyanate groups of isocyanate-terminated polybutadiene (ITPB) is depicted in Figure 2. The isocyanate groups feature a conjugated unsaturated structure that exhibits high chemical reactivity with various hydrogen-containing compounds. This high reactivity facilitates the reaction between ITPB and the hydroxyl groups on the silica surface, thereby reducing the silica’s hydrophilicity and preventing agglomeration, which enhances dispersion. Following the reaction, the isocyanate groups at both ends of the ITPB molecular chain formed ‘chemical bridges’ between the silica particles, creating a physical barrier that further inhibits silica agglomeration.

#### 3.1.2. Thermogravimetric Analysis (TGA)

**Figure 3:**
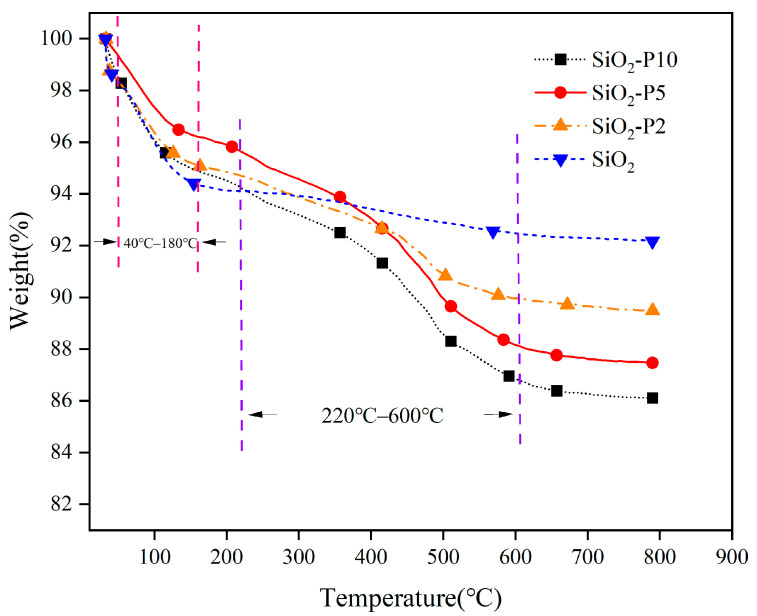
Thermogravimetric analysis (TGA) curve of ITPB-modified silica.

Figure 3 illustrates the thermal weight loss profiles of both modified and unmodified silica. The primary weight loss occurs in two distinct temperature regions: 40–180 °C and 220–600 °C. In the 40–180 °C region, both types of silica exhibit weight loss, predominantly due to the volatilization of hydroxyl groups and adsorbed water from the silica surface. Notably, unmodified silica demonstrated significantly greater weight loss compared to the modified silica within this range. This difference can be attributed to the reduction of hydroxyl groups on the silica surface during the modification process with liquid rubber, which consequently decreases the weight loss observed during heating for the modified silica. In the 220–600 °C region, modified silica showed more weight loss compared to unmodified silica. This additional weight loss is attributed to the decomposition of methylene groups present in the liquid rubber, in addition to the loss of hydroxyl groups. In contrast to unmodified silica, modified silica displayed two clear weight loss stages, each correlating with the weight loss of the soft and hard segments of ITPB. This observation confirms the successful modification of silica with liquid rubber, indicating the effective grafting of ITPB onto the silica surface. The grafting percentage of ITPB can be determined via thermogravimetric analysis (TGA) using the formula provided in Equation (1).

Grafting efficiency of SiO_2_:

(1) $$Grafting\;percentages=\frac{∆\omega_{CH}}{\omega_{{SiO}_{2}}+∆\omega_{{SiO}_{2}}(40\sim 220\;{}^{\circ}C)+∆OH}\times 100\%$$

where $∆\omega_{CH}=∆\omega_{{SiO}_{2}}\left(220\sim 600\;{}^{\circ}C\right)-∆OH$ represents the mass loss of liquid rubber grafted onto modified silica. $\omega_{{SiO}_{2}}$ denotes the residue of modified SiO_2_ after TGA testing, $∆\omega_{{SiO}_{2}}(40\sim 220\;{}^{\circ}C)$ signifies the sum of the water and hydroxyl group losses from the silica surface, and ∆OH indicates the hydroxyl group loss from SiO_2_ in the 220–600 °C range.

By calculating the grafting percentage, the mass of liquid rubber grafted onto silica can be determined, which subsequently enables the calculation of the grafting efficiency of the silica, as detailed in Table 3. It is observed that as the amount of ITPB liquid rubber increases, the grafting percentage of silica also increases. However, the grafting efficiency gradually decreases, which is attributed to the steric hindrance effect caused by the rubber molecules. As each rubber molecule attaches to the surface, it occupies space and reduces the available area for additional grafting on the silica, thereby impeding the further attachment of ITPB to rubber molecules and diminishing the overall grafting efficiency.

#### 3.1.3. Surface Area Analysis

The BET surface area of the modified silica was measured using the BET-ASAP2460 surface area analyzer (Micromeritics, Norcross, GA, USA); the results are presented in Table 3. As shown, the BET surface area of the modified silica is significantly smaller compared to that of the unmodified silica. The specific surface area (SSA) values of the modified silica decrease progressively with increasing amounts of ITPB liquid rubber, with reductions of 19.45%, 23.81%, and 27.78% respectively. This decrease is attributed to the increased number of molecular chains grafted onto the silica surface, which reduces the available area for nitrogen adsorption and consequently leads to reductions in the specific surface area. This observation aligns with the grafting rates obtained from TGA analysis.

### 3.2. Tests of ITPB-Modified Silica for Enhanced SBR

#### 3.2.1. Curing Characteristics

**Figure 4:**
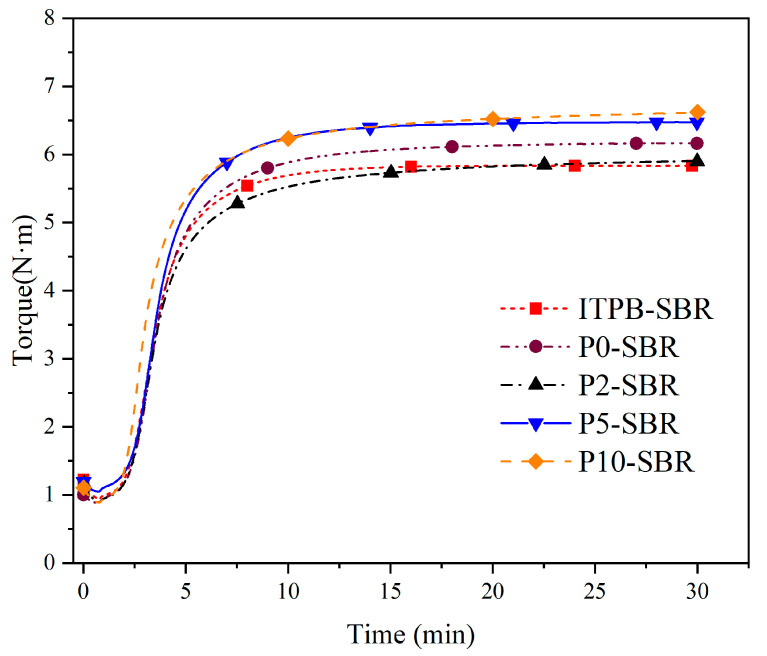
Vulcanization characteristics of enhanced SBR composites.

Figure 4 presents the curing curves of rubber samples prepared at the same dosage using modified silica with different grafting ratios, and the relevant curing parameters are listed in Table 4. As indicated in Table 2, sample P0-SBR represents rubber compounded with unmodified silica, while ITBP-SBR refers to rubber compounded with a physical blend of liquid rubber and unmodified silica. Samples P2-SBR, P5-SBR, and P10-SBR represent rubber compounded with silica modified by liquid rubber at different, progressively increasing, grafting rates.

From Table 4, it is evident that both the blending modification and chemical modification of silica fillers can lead to an increase in t_S2_ and a decrease in t_90_ during the vulcanization process. The increase in t_S2_ indicates a delay in cross-linking, thereby broadening the processing window for rubber compounding and enhancing processing safety. Concurrently, the decrease in t_90_ facilitates a reduction in vulcanization time. This is because the modification lowers the surface adsorption rate of silica, reducing its interaction with vulcanization accelerators and thus compensating for the delayed vulcanization inherent to silica. Liquid rubber, when directly added, reacts with sulfur during mixing, leading to premature cross-linking and a reduced vulcanization time. Additionally, ITPB reacts with silica during mixing, accelerating the vulcanization rate. For chemically modified silica-filled rubber, an increase in grafted liquid rubber improves t_S2_ and t_90_ more effectively, correlating with a greater reduction in silica’s surface adsorption rate.

Compared to samples with directly added silica, the incorporation of ITBP increases the interaction between silica and rubber, resulting in a rise in M_L_ for all systems relative to P0-SBR. This is due to the higher viscosity of ITBP, which decreases the processing flowability. However, the M_H_-M_L_ value for rubber with blended silica modifications decreased compared to P0-SBR, as the direct addition of liquid rubber hinders the formation of a cross-linking network, leading to lower cross-link density. For chemically modified silica-filled rubber, P2-SBR exhibited a reduction, while the other two samples showed increased values, which positively correlated with ITBP content, indicating that grafted liquid rubber participated in the vulcanization process.

Overall, P5-SBR and P10-SBR demonstrate the best processing performance and mechanical strength. Upon observing their vulcanization curves, it is apparent that the sample with an ITPB-to-silica ratio of 1:20 has a relatively flat vulcanization curve, indicating a dynamic equilibrium between the formation and breakage of cross-linking, which is most favorable for the vulcanization process and preserves the sample’s performance. Increasing the ITPB-to-silica ratio to 1:10 results in a curve that first levels off and then gradually increases with extended vulcanization time, suggesting that the formation rate of the cross-linking surpasses its breakage, thus increasing cross-link density. Therefore, the ideal ratio of ITPB to silica is 1:20.

#### 3.2.2. Thermal Stability Analysis

**Figure 5:**
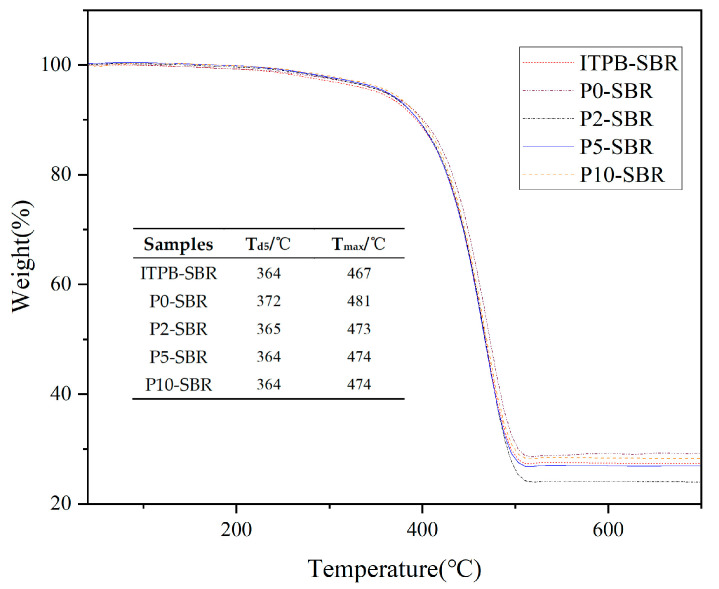
Thermogravimetric profiles of BR composites.

Figure 5 illustrates the TGA curves of rubber samples prepared with varying proportions of modified silica. The TGA data indicate that as the temperature rises from 40 °C to 200 °C, the samples experience a weight loss of 0.2% to 0.5%, primarily due to the evaporation of trace moisture inherent in the rubber. From 200 °C to 300 °C, a gradual but negligible weight loss occurs, likely from volatile substances within the rubber matrix. Notably, from 350 °C to 550 °C, a pronounced and rapid decline in weight is observed, indicating significant thermal degradation of the polymer’s chain backbone. The mass loss at 5% (Td5) and the temperature corresponding to the maximum mass loss rate (Tmax) are critical factors for assessing the thermal stability of rubber composites. The Td5 values for ITPB-SBR, P0-SBR, P2-SBR, P5-SBR, and P10-SBR are 364 °C, 372 °C, 365 °C, 364 °C, and 364 °C, respectively, all significantly exceeding the processing and operational temperatures of the rubber samples, confirming their adequate thermal stability. The Tmax values for these composites are 474 °C, 481 °C, 473 °C, 474 °C, and 474 °C, respectively, suggesting that the introduction of ITPB does not significantly impact the thermal stability of SBR, as evidenced by the similarity in Tmax values between ITPB-modified silica-filled rubber and P0-SBR.

#### 3.2.3. Mechanical Properties

**Figure 6:**
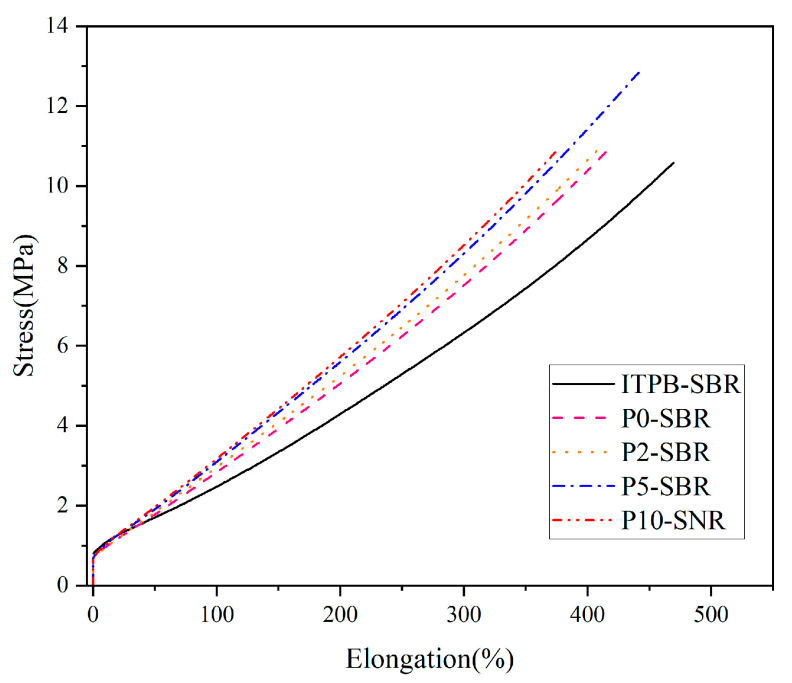
The tensile stress–strain curve of the SBR composites.

Based on the characteristics of the rubber vulcanization process observed, we developed corresponding vulcanization parameters for each formulation. Rubber compounds with varying compositions were placed into a hot-press molding machine and vulcanized at 160 °C, with the vulcanization time controlled to t_90_ + 3 min. Rubber samples with different performances were produced. The samples were subjected to stress–strain testing using a universal testing machine at a rate of 500 mm/min, and Shore hardness was measured with a Shore hardness tester, as detailed in Table 5. The hardness test results indicate that the Shore hardness of the rubber progressively increased with the addition of liquid rubber, which was attributed to the increased degree of vulcanization upon the addition of liquid rubber. A higher vulcanization degree generally leads to a denser cross-linking network and a consequently higher hardness. The results of the rubber tensile modulus and elongation at break indicate that the tensile modulus of the ITPB-modified silica-filled rubber exhibits little variation. In contrast, the direct addition of ITPB reduced the tensile modulus of the rubber. This was attributed to the plasticizing effect of ITPB during processing, which enhances the elasticity of the resulting rubber. Figure 6 presents the stress–strain curve of the rubber during tensile testing. Tensile testing revealed that the tensile strength of rubber that directly incorporated ITPB decreased compared to the control sample without ITPB. This reduction was due to the reduced interaction between rubber and filler in the ITPB-SBR sample, suggesting that direct addition of ITPB does not enhance tensile performance. In the case of rubber modified with precipitated silica, the tensile strength of P10-SBR decreased relative to the control sample. This decline was attributed to the excessive presence of flexible ITPB molecular chains, which causes silica particles to agglomerate and reduces the reinforcing effect of silica on the rubber. Conversely, insufficient ITPB fails to adequately modify silica, leading to minimal reinforcement improvements. P5-SBR rubber exhibited a tensile strength of 12.6 MPa, representing an 11.5% improvement over the unmodified silica-filled rubber. This enhancement is due to the optimal ratio of ITPB to silica (1:20), which provides the best dispersion of silica. The reduced number of stress concentration zones due to the reduced chemical interface interactions between silica particles enhances the reinforcing efficiency of silica, yielding the best reinforcement effect for the rubber.

**Figure 7:**
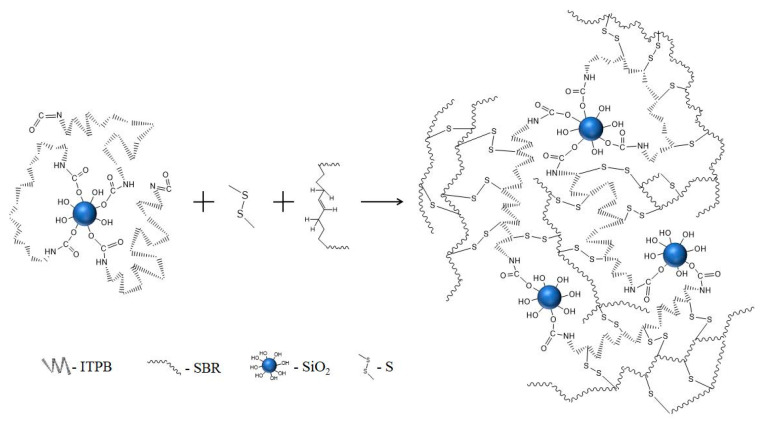
Schematic diagram of crosslinking reactions between chemically modified silica and rubber.

As illustrated in Figure 2, the physical shielding effect of the long polymer chains effectively prevents grafted silica particles from agglomerating with other silica particles, thereby enhancing their dispersion. However, the flexible and elastic nature of ITPB molecules can also lead to the formation of a silica network, where some grafted silica particles may connect, resulting in slightly larger agglomerate sizes. Additionally, as shown in Figure 7, the double bonds present in the grafted ITPB on silica participate in the rubber vulcanization process. This involvement strengthens the chemical interactions between the silica and SBR rubber, ultimately improving the mechanical strength of the composite [28].

**Figure 8:**
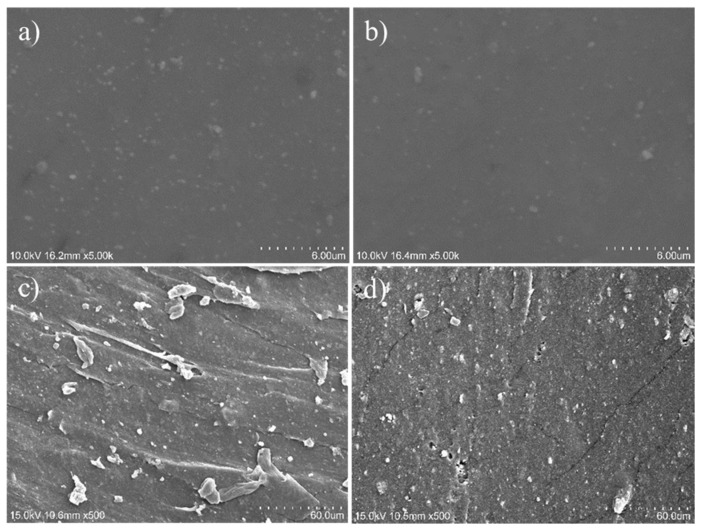
(**a**) SEM image of P0-SBR rubber; (**b**) SEM image of P5-SBR rubber; (**c**) fracture surface of P0-SBR rubber; (**d**) fracture surface of P5-SBR rubber.

The results of scanning electron microscopy (SEM) further confirm the synergistic effects of modified silica. Figure 8a,b presents SEM images of rubber filled with silica, where the white areas indicate agglomerates of silica. The larger the agglomerates, the poorer the dispersion of silica within the SBR matrix. In Figure 8a, the silica agglomerates are notably larger, indicating poor dispersion in P0-SBR. In contrast, Figure 8b shows a significant reduction in the size and quantity of agglomerates for modified silica, demonstrating that the modification with ITPB improved the dispersion of silica. Figure 8c,d illustrates the tensile fracture surfaces of silica-filled rubber. Figure 8c shows a relatively smooth fracture surface with large rubber particles, indicating weak interaction between the rubber and filler, leading to stress concentration during stretching. In Figure 8d, the fracture surface of P5-SBR is rougher, which can be attributed to the improved dispersion of silica. This results in a more uniform stress distribution at the rubber interface during stretching, leading to an enhanced mechanical performance compared to P0-SBR.

The enhancement of the mechanical properties of chemically modified silica-filled SBR can also be validated by the changes in cross-link density. The cross-link density can be calculated using the Flory–Rehner equation through equilibrium swelling analysis. The cross-link density formula [35] is as follows:

(2) $$v_{e}=-\frac{\ln\left(1-v_{r}\right)+v_{r}+x{v_{r}}^{2}}{V_{s}({v_{r}}^{1/3}-0.5v_{r})}$$

where $v_{e}$ denotes the cross-linking density of SBR, expressed in units of mol/cm^3^. $v_{e}$ represents the molar volume of the solvent and $v_{s}$ denotes the volume fraction of soluble rubber after swelling. The formula [36] is as follows:

(3) $$v_{r}=\frac{m_{2}/\rho_{r}}{(m_{1}-m_{2})/\rho_{s}+m_{2}/\rho_{r}}$$

Here, $m_{3}=m_{1}-m_{2}$ represents the mass of insoluble components in the sample $\rho_{r}$ denotes the density of the vulcanized rubber, $\rho_{s}$ denotes the density of the solvent, and x represents the interaction coefficient between rubber and the solvent xylene. The formula is given as follows:

(4) $$x=\frac{V_{s}{\left(\delta_{r}-\delta_{s}\right)}^{2}}{R\;T}+0.34$$

Here, $\delta_{r}$ denotes the solubility parameter of SBR, which is 8.3 (J/cm^3^)^1/2^, while $\delta_{s}$ represents the solubility parameter of the solvent, which is 8.8 (J/cm^3^)^1/2^.

The results of the cross-link density calculations are presented in Table 5. It is evident that using liquid rubber to modify silica increases the rubber’s cross-link density, and this density increases according to the amount of liquid rubber that is added, indicating that more cross-linking points are formed. Although the direct addition of liquid rubber to physically blended silica-filled rubber improves flowability, the shorter molecular chains of the liquid rubber are not conducive to the formation of cross-linking networks, resulting in a lower cross-link density compared to P0-SBR. In contrast, for chemically modified fillers, functionalized liquid rubber that is pre-grafted onto the silica surface is mainly concentrated at the interface between silica and the rubber matrix. This approach does not disrupt the intrinsic cross-linking but enhances the compatibility and interaction between silica and rubber, thereby improving both cross-link strength and density.

#### 3.2.4. Dynamic Mechanical Thermal Performance Analysis

**Figure 9:**
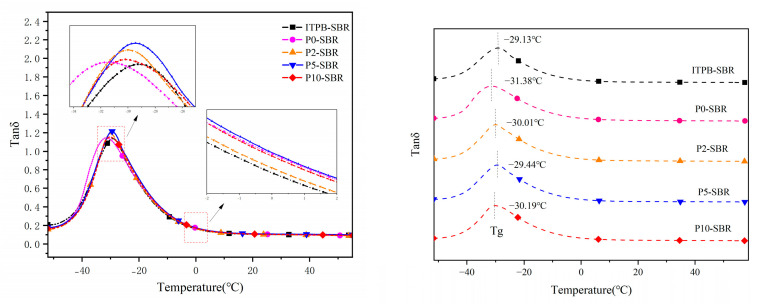
Temperature curve of Tan δ for rubber.

Figure 9 depicts the temperature-dependent tanδ curves of vulcanized rubber under 0.1% strain, allowing for an investigation of the interfacial effects of silica on the movement of rubber chains. Generally, the peak value of tanδ reflects the mobility of rubber’s molecular chains. After the formation of a filler network, the aggregation of fillers restricts the motion of rubber chains. The decrease in the peak height corresponds to the reduction in the amount of rubber that does not participate in the glass transition process. Thus, the peak value of tan δ also indirectly indicates the dispersion of fillers within the rubber matrix. When fillers are uniformly dispersed, the restriction on rubber chains caused by filler–filler interactions is minimized, leading to an increased number of effective rubber chains and a corresponding rise in the glass transition temperature, as indicated by tanδ_max_ [37].

For rubber composites filled with silica modified by varying amounts of ITPB, P5-SBR exhibits a significantly higher tanδ_max_ compared to unmodified rubber, indicating that ITPB improves the filler network and enhances silica dispersion. However, P10-SBR shows a marked decrease in tanδ_max_ compared to P5-SBR, due to the excessive grafting of ITPB molecules, resulting in excessive cross-linking between fillers. When comparing the tanδ_max_ of rubber filled with directly added ITPB-modified silica to that of grafted and modified silica, the latter shows a higher tanδ_max_. This is attributed to the fact that grafted rubber chains better prevent silica agglomeration, whereas the direct addition of ITPB does not significantly improve silica dispersion.

As illustrated in Figure 9, the addition of ITPB leads to an increase in the glass transition temperature of the rubber products owing to the increased number of cross-linking points. tanδ values also serve to evaluate the wet skid resistance and rolling resistance of rubber composites. A higher tanδ (0 °C) indicates better wet skid resistance [38,39], while a higher tanδ (60 °C) reflects greater rolling resistance [40,41]. P0-SBR rubber shows the highest tanδ (0 °C), suggesting that the use of ITPB does not enhance the wet skid resistance. Conversely, the tanδ (60 °C) of rubber modified with ITPB increases relative to P0-SBR, indicating that while ITPB improves silica dispersion, it also introduces numerous free end chains, thereby increasing the rolling resistance of the rubber.

## 4. Conclusions

This study involved the preparation of graft-modified silica with varying ITPB contents using toluene as the solvent through heating and stirring. The modified silica was then used to fill the SBR rubber matrix, producing chemically modified silica-filled rubber composites. These composites were compared with unmodified silica-filled rubber and physically blended silica-filled rubber to investigate the impact of different modification methods and ITPB quantities on the dispersion of silica in butadiene–styrene rubber, the silica–rubber interface structure, and the rubber’s overall properties. Compared to physical blending modifications, chemical modifications significantly enhance the dispersion of silica and the strength of the interface interaction, resulting in superior static and dynamic mechanical properties for the rubber composites. When using chemically modified silica, the tensile strength first increases and then decreases with increasing ITPB content. This behavior is attributed to excessive cross-linking and the agglomeration of silica due to the ITPB molecular chains. The optimal performance was achieved with an ITPB-to-silica ratio of 1:20, where the tensile strength improved by 11.5%. This highlights the potential applications of this rubber material in rail transportation and automotive tires.

## Figures and Tables

**Table 1:** Designation and corresponding feed ratios of ITPB-modified silica particles.

Sample	m(ITPB):m(Silica)
SiO_2_-P2	2:100
SiO_2_-P5	5:100
SiO_2_-P10	10:100

**Table 2:** Compounding formulation of modified silica-filled SBR composites (units: phr).

Sample	ITPB-SBR	P0-SBR	P10-SBR	P5-SBR	P2-SBR
SBR	100	100	100	100	100
Silica	25	25	--	--	--
ITPB	1.5	--	--	--	--
SiO_2_-P10	--	--	25	--	--
SiO_2_-P5	--	--	--	25	--
SiO_2_-P2	--	--	--	--	25
ZnO	5	5	5	5	5
SAA	2	2	2	2	2
774	20	20	20	20	20
S	1	1	1	1	1
DM	2	2	2	2	2
TMTD	1	1	1	1	1

**Table 3:** Grafting efficiency of ITPB-modified silica.

Sample	Grafting Ratio	Grafting Efficiency	SSA (cm^2^/g)
SiO_2_	--	--	170.7 ± 1.4
SiO_2_-P2	1.71%	85.5%	137.3 ± 1.3
SiO_2_-P5	3.63%	72.6%	129.7 ± 1.2
SiO_2_-P10	6.30%	63.0%	123.3 ± 1.2

**Table 4:** Vulcanization characteristics of enhanced SBR composites.

Sample	t_S2_/min	t_90_/min	M_L_/N·m	M_H_/N·m	M_H_-M_L_/N·m
P0-SBR	1:34	7:40	0.838	6.172	5.334
ITPB-SBR	1:44	6:46	0.933	5.845	4.912
P2-SBR	1:36	7:20	0.883	5.911	5.028
P5-SBR	1:38	7:15	1.006	6.484	5.478
P10-SBR	1:39	6:48	0.888	6.628	5.740

**Table 5:** Mechanical properties and crosslink density of the rubber.

Sample	Tensile Strength/MPa	Fracture Strength/N	Elongation at Break/%	Tensile Modulus/MPa	Shore Hardness	Crosslink Density/10^−4^ mol/cm^3^
P0-SBR	11.3	169.2	412.1	0.027	62	2.01
ITPB-SBR	10.8	135.6	469.5	0.023	64	1.70
P2-SBR	11.2	157.3	420.9	0.027	64	2.08
P5-SBR	12.6	185.0	441.5	0.028	65	2.15
P10-SBR	10.5	154.80	374.9	0.028	66	2.23

## Data Availability

All raw data are available on request.
